# A Hormonally Active Malignant Struma Ovarii

**DOI:** 10.1155/2016/2643470

**Published:** 2016-11-02

**Authors:** Carolina Lara, Dalia Cuenca, Latife Salame, Rafael Padilla-Longoria, Moisés Mercado

**Affiliations:** ^1^Department of Medicine, ABC Medical Center, Mexico City 01120, Mexico; ^2^Neurological Center, ABC Medical Center, Mexico City 01120, Mexico; ^3^Department of Surgical Oncology, Hospital Angeles de Interlomas, Huixquilucan 52763, Mexico

## Abstract

Struma ovarii is a rare monodermal variant of ovarian teratoma that contains at least 50% thyroid tissue. Less than 8% of struma ovarii cases present with clinical and biochemical evidence of thyrotoxicosis due to ectopic production of thyroid hormone and only 5% undergo malignant transformation into a papillary thyroid carcinoma. Only isolated cases of hormonally active papillary thyroid carcinoma developing within a struma ovarii have been reported in the literature. We report the case of a 36-year-old woman who presented with clinical signs and symptoms of hyperthyroidism as well as a left adnexal mass, which proved to be a thyroid hormone-producing, malignant struma ovarii.

## 1. Introduction

Germ cell tumors represent 15–20% of all ovarian cancers and most of them are mature cystic teratomas [1]. Struma ovarii (SO) is a monodermal variant of ovarian teratoma that contains at least 50% of thyroid tissue [1]. Initially described by Buttlin in 1888, SO was first recognized to consist of thyroid tissue in 1902 by Pick [2]. In approximately 5% of cases, the thyroid tissue within SO is found to contain areas of well-differentiated carcinoma, most commonly, papillary thyroid carcinoma (PTC) [3, 4]. SO generally occurs in women in the fifth and sixth decades of life, presenting in the majority of cases, as a unilateral adnexal mass, with clear left side predominance [3–5]. Over 92% of the patients are clinically and biochemically euthyroid [5–7]. Thus, the occurrence of hyperfunctioning thyroid tissue within SO is a very rare event and is usually listed in the differential diagnosis of thyrotoxicosis in the setting of a low radioiodine uptake [5–7]. We here in present the case of a woman with a pelvic mass that proved to be a PTC containing, hormonally active SO.

## 2. Case Report

A previously healthy, 36-year-old woman was admitted to the hospital because of left lower abdominal pain and nausea of 5 days' duration. Pain was referred to as intense, colicky, and located over the left iliac fossa and radiating to her left groin and lower back. She denied fever, chills, genitourinary symptoms, or any changes in her bowel habits but mentioned palpitations, heat intolerance, a 7-Kg unintentional weight loss, insomnia, and tremor over the preceding 6 months. On physical exam, her blood pressure was 105/60 mmHg, heart rate 110 bpm and regular, respiratory rate 20, and temperature 36.5°C. She appeared notoriously anxious but in no acute distress. Cardiopulmonary exam revealed a hyperdynamic precordium. Neck was supple; the thyroid gland was not readily palpable. The abdomen was soft, with no palpable organomegalies; she had pain over the left iliac fossa upon deep palpation with no rebound tenderness and the peristalsis was normal. She has had two previous uneventful pregnancies, the latest one three years prior to admission; her menstrual cycles were regular and she did not use any contraceptive method. She had no allergies, did not use any medications, and regularly abused alcohol on weekends (usually a pint of tequila). Her family history was remarkable for primary hypothyroidism in her maternal grandmother.

**Figure 1:**
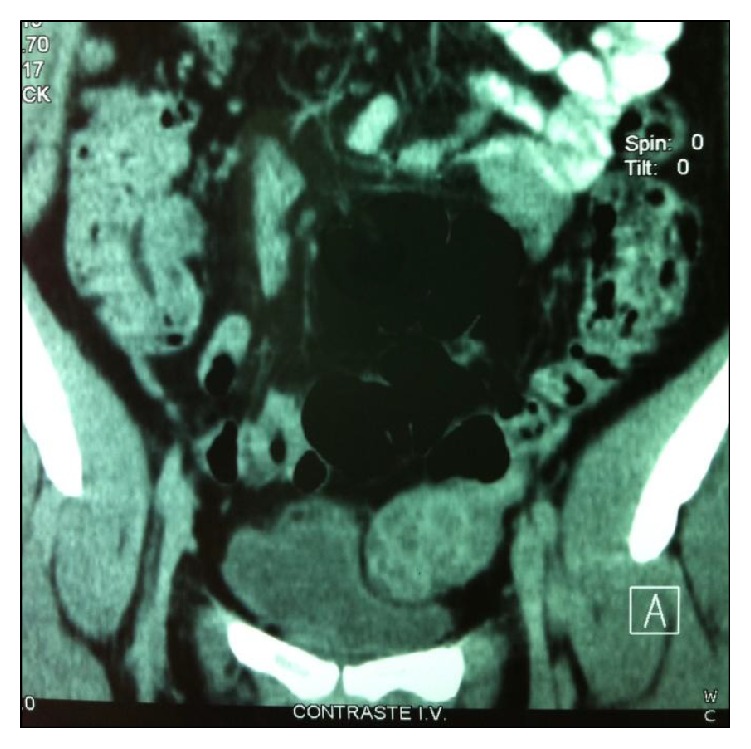
Contrast-enhanced abdominal CT revealing tumour on left ovary.

Initial laboratory workup showed a normal CBC as well as basic blood chemistry, liver function tests, serum electrolytes, and urinalysis. Thyroid function tests revealed a TSH of <0.01 mIU/mL and a free T4 of 2.6 ng/dL. A solid, left adnexal mass was found on abdominal-pelvic ultrasound and was confirmed by CT scan (Figure 1). A ^99m^Tc thyroid scan showed a complete absence of uptake. Thyroid ultrasonography was unrevealing.

**Figure 2:**
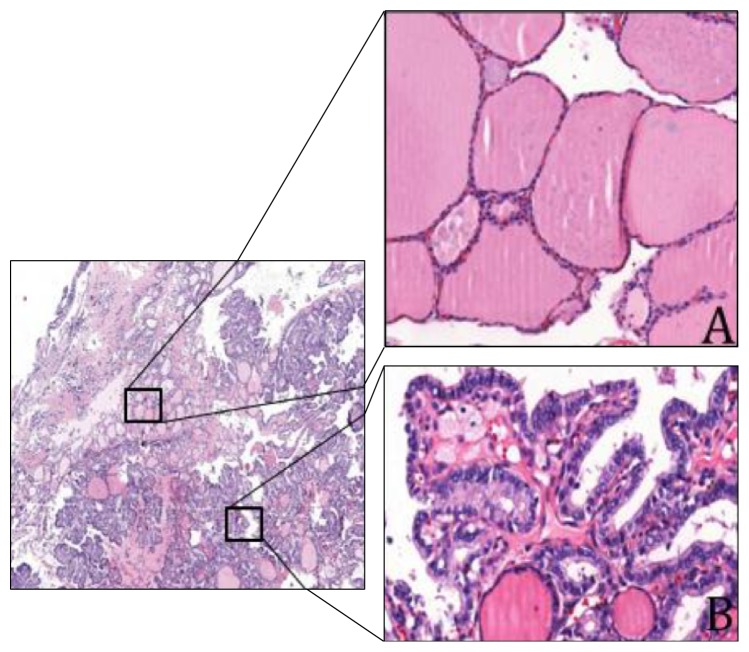
Malignant struma ovarii with classic variant papillary thyroid cancer. On (A) normal thyroid tissue and (B) areas with papillary thyroid cancer.

**Figure 3:**
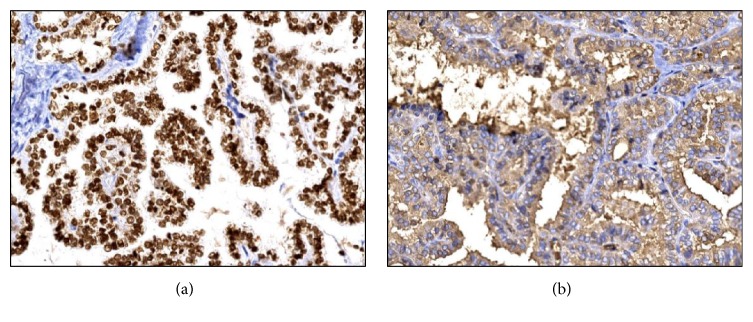
Positive immunohistochemical stain for TTF-1 (a) and thyroglobulin (b).

Upon surgical exploration she was found to have a solid 50 × 40 × 30 mm left ovarian mass and a left salpingo-oophorectomy with omentectomy was performed. On histopathological examination the mass consisted of normal thyroid tissue with several areas of papillary carcinoma with multifocal capsular invasion but without perineural or vascular extension (Figure 2). Immunohistochemistry was strongly positive for both thyroid transcription factor-1 (TTF-1) and thyroglobulin (Figure 3).

In view of these findings, it was decided to proceed with a total thyroidectomy. On histopathological examination the thyroid gland appeared completely normal. Four weeks later she received a 75 mCi, ablative radioactive iodine dose. Thyroid bed uptake was documented upon a posttherapeutic total body scan, with no abnormal radioiodine accumulation anywhere else.

The patient is currently asymptomatic on thyroid suppression therapy; her latest recombinant TSH-stimulated thyroglobulin was undetectable one year after initial presentation.

## 3. Discussion

The most common clinical presentation of SO is that of a pelvic mass, which can either be incidentally found on pelvic ultrasound or manifest as lower abdominal pain [3–5]. Less than 8% of the patients with SO present with hyperthyroidism and when they do, it is usually mild and subclinical [5–7]. Our patient presented with lower abdominal pain and clinical signs and symptoms, as well as biochemical evidence of thyrotoxicosis. In fact, a hormonally active SO should be considered in the differential diagnosis of thyrotoxicosis in the setting of a low or absent radioiodine or tecnetium thyroid uptake [5–7]. The pathogenesis of thyrotoxicosis in patients with SO is incompletely understood. There have been several case reports of patients with preexisting or concomitant Graves' disease in whom anti-TSH receptor autoantibodies stimulate thyroid hormone production in both the eutopic and ectopic thyroid tissues; in these cases the diagnosis of SO has been made only after an apparently successful thyroid ablation fails to normalize thyroid hormone levels [8, 9]. Unfortunately thyroid autoantibodies were not available in our patient, although she did not have any features suggestive of autoimmune thyroid disease such as Graves' ophthalmopathy. Somatic, activating mutations of the TSH receptor explain the hyperthyroidism in the majority of autonomous thyroid nodules and such mutations have been sought but not found in patients with thyrotoxicosis associated with SO [7]. Thus, it appears that in most patients with hyperthyroidism due to SO the autonomous production of thyroid hormone by the ectopic tissue is not related to the presence of TSH receptor antibodies or to gain-of-function mutations of the TSH receptor but to mechanisms more closely resembling those involved in Plummer's disease or toxic multinodular goiter.

The emergence of a well-differentiated carcinoma arising in these ectopically located thyroid tissues is an infrequent event, occurring in about 5% of all SO cases [3, 10, 11]. Although all the variants of well-differentiated thyroid cancer have been reported, in over 70% of the cases the histopathology is that of a typical papillary thyroid carcinoma [3, 10–12]. Immunohistochemically these tumors stain positive for both thyroglobulin and TTF-1 [10]. The possibility of a primary papillary thyroid carcinoma with ovarian metastases needs to be ruled out, which is achieved by demonstrating the absence of thyroid lesions by means of high-resolution ultrasonography, CT, or even MRI. Multiple molecular abnormalities have been described in thyroid cancers arising from ovarian teratomas, including point mutations in* BRAF* (V600E and K601E) 67% of cases [13] and* KRAS* and* NRAS* [13] as well as loss of heterozigocity in the PTEN region and* ret/PTC* rearrangements [12, 13].

There are no consensus guidelines for the management of malignant SO. In patients who wish to preserve fertility and provided the disease burden is confined to the ovary the recommended approach consists of unilateral salpingo-oophorectomy, along with near-total thyroidectomy and radioactive iodine ablation [10, 14]. When fertility is not an issue and/or in cases with more invasive tumors, a total abdominal hysterectomy with bilateral salpingo-oophorectomy is the usual course of action; adjunctive systemic chemotherapy has been used in patients with distant metastasis. As was done in our case, some authors recommend thyroidectomy and ^131^I ablation as the first-line of treatment in order to be able to use the measurement of thyroglobulin as a tumor marker [3, 15, 16].

## 4. Conclusion

Struma ovarii is a monodermal variant of ovarian teratoma, defined by the presence of >50% thyroid tissue that generally occurs in women between the ages of 40 and 60 and usually presents as a unilateral adnexal mass. The occurrence of hyperfunctioning thyroid tissue within struma ovarii is a very rare event. When fertility is not a concern and in patients with invasive tumors, treatment consists of total abdominal hysterectomy with bilateral salpingo-oophorectomy; adjuvant chemotherapy is recommended when distant metastasis is present. In patients who wish to preserve fertility and provided the tumor is confined to one ovary, the recommended treatment is unilateral salpingo-oophprectomy, along with total thyroidectomy and radioactive iodine ablation.

## References

[1] Roth L. M., Talerman A. (2007). The enigma of struma ovarii. *Pathology*.

[2] Pick L. (1903). Beitrag zur Lehre von den Greschwülsten über struma thyroidaea ovarii aberrata. *Verhandlungen der Berliner Medizinischen Geselschaft*.

[3] Yassa L., Sadow P., Marqusee E. (2008). Malignant struma ovarii. *Nature Clinical Practice Endocrinology and Metabolism*.

[4] Goffredo P., Sawka A. M., Pura J., Adam M. A., Roman S. A., Sosa J. A. (2015). Malignant struma ovarii: a population-level analysis of a large series of 68 patients. *Thyroid*.

[5] Yoo S. C., Chang K. H., Lyu M. O., Chang S. J., Ryu H. S., Kim H. S. (2008). Clinical characteristics of struma ovarii. *Journal of Gynecologic Oncology*.

[6] Matsuda K., Maehama T., Kanazawa K. (2001). Malignant struma ovarii with thyrotoxicosis. *Gynecologic Oncology*.

[7] Ciccarelli A., Valdes-Socin H., Parma J. (2004). Thyrotoxic adenoma followed by atypical hyperthyroidism due to struma ovarii: clinical and genetic studies. *European Journal of Endocrinology*.

[8] Mimura Y., Kishida M., Masuyama H. (2001). Coexistence of Graves' disease and struma ovarii: case report and literature review. *Endocrine Journal*.

[9] Sitasuwan T., Hanamornroongruang S., Peerapatdit T., Thongtang N. (2015). Coexistence of Graves' disease and unilateral functioning struma ovarii: a case report. *BMC Endocrine Disorders*.

[10] Zhang X., Axiotis C. (2010). Thyroid-type carcinoma of struma ovarii. *Archives of Pathology and Laboratory Medicine*.

[11] Roth L. M., Miller A. W., Talerman A. (2008). Typical thyroid-type carcinoma arising in struma ovarii: a report of 4 cases and review of the literature. *International Journal of Gynecological Pathology*.

[12] Boutross-Tadross O., Saleh R., Asa S. L. (2007). Follicular variant papillary thyroid carcinoma arising in struma ovarii. *Endocrine Pathology*.

[13] Schmidt J., Derr V., Heinrich M. C. (2007). BRAF in papillary thyroid carcinoma of ovary (struma ovarii). *The American Journal of Surgical Pathology*.

[14] Jean S., Tanyi J. L., Montone K., McGrath C., Lage-Alvarez M. M., Chu C. S. (2012). Papillary thyroid cancer arising in struma ovarii. *Journal of Obstetrics and Gynaecology*.

[15] DeSimone C. P., Lele S. M., Modesitt S. C. (2003). Malignant struma ovarii: a case report and analysis of cases reported in the literature with focus on survival and I131 therapy. *Gynecologic Oncology*.

[16] Pacini F., Schlumberger M., Harmer C. (2005). Post-surgical use of radioiodine (^131^I) in patients with papillary and follicular thyroid cancer and the issue of remnant ablation: a consensus report. *European Journal of Endocrinology*.

